# Partitioned polygenic risk scores identify distinct types of metabolic dysfunction-associated steatotic liver disease

**DOI:** 10.21203/rs.3.rs-3878807/v1

**Published:** 2024-02-06

**Authors:** Stefano Romeo, Oveis Jamialahmadi, Antonio De Vincentis, Federica Tavaglione, Francesco Malvestiti, Ruifang Li-Gao, Rosellina Mancina, Marcus Alvarez, Kyla Gelev, Samantha Maurotti, Umberto Vespasiani-Gentilucci, Frits Rosendaal, Julia Kozlitina, Päivi Pajukanta, François Pattou, Luca Valenti

**Affiliations:** Department of Molecular and Clinical Medicine, Institute of Medicine, Sahlgrenska Academy, Wallenberg Laboratory, University of Gothenburg; University of Gothenburg; Operative Unit of Internal Medicine, Fondazione Policlinico Universitario Campus Bio-Medico, Rome, Italy; University of Gothenburg; University of Milan; Leiden University Medical Center; Department of Molecular and Clinical Medicine, Institute of Medicine, Sahlgrenska Academy, Wallenberg Laboratory, University of Gothenburg; University of California, Los Angeles; David Geffen School of Medicine at UCLA; Magna Graecia University; Fondazione Policlinico Universitario Campus Bio-Medico; Leiden University Medical Center; UT Southwestern; University of California, Los Angeles; Centre Hospitalier Universitaire de Lille; University of Milan

## Abstract

Metabolic dysfunction-associated steatotic liver disease (MASLD) encompasses an excess of triglycerides in the liver, which can lead to cirrhosis and liver cancer. While there is solid epidemiological evidence of MASLD coexisting with cardiometabolic disease, several leading genetic risk factors for MASLD do not increase the risk of cardiovascular disease, suggesting no causal relationship between MASLD and cardiometabolic derangement.

In this work, we leveraged measurements of visceral adiposity and identified 27 novel genetic *loci* associated with MASLD. Among these *loci,* we replicated 6 in several independent cohorts. Next, we generated two partitioned polygenic risk scores (PRS) based on the mechanism of genetic association with MASLD encompassing intra-hepatic lipoprotein retention. The two PRS suggest the presence of at least two distinct types of MASLD, one confined to the liver resulting in a more aggressive liver disease and one that is systemic and results in a higher risk of cardiometabolic disease.

## Introduction

Paralleling the obesity epidemic, metabolic dysfunction-associated steatotic liver disease (MASLD) is a growing burden worldwide. MASLD is a spectrum of conditions encompassing an excess of triglycerides in the liver progressing to inflammation, fibrosis and ultimately to cirrhosis and liver cancer^1^. MASLD is a heterogenous disease that epidemiologically coexists with a metabolic derangement, including visceral adiposity, insulin resistance, dyslipidemia, hypertension and hyperglycemia. This metabolic derangement ultimately increases the risk of cardiovascular events including heart failure and kidney disease^2–4^. Indeed, cardiovascular disease is the most frequent cause of death in individuals with MASLD, whereas hepatic failure leading to liver-related events is a less frequent complication. However, it is a common clinical observation, yet to be understood, that some individuals develop a severe and rapidly progressing liver disease despite similar or even less marked metabolic derangement.

MASLD has a strong inherited component, with variants that increase primarily liver triglyceride accumulation by impairing hepatocyte lipid droplet remodeling and lipoprotein secretion involved in its development and progression^5^. However, contrarily to the epidemiological evidence, these variants result in a protection against cardiovascular disease and no association with hypertension^5–7^ and heart failure, suggesting no causal relationship between MASLD and cardiometabolic derangement^5^.

Over the last 15 years, genome-wide association studies (GWAS) identified several genetic *loci* associated with chronic liver disease or proxies for increased liver triglyceride content^8–13^. Studies have also shown that, excess in adiposity amplifies the effect size of these variants^14^ likely by increasing ectopic visceral fat. However, anthropometric measures of adiposity (body mass index, BMI) and body fat distribution (waist circumference, WC) fail to provide an accurate quantification of visceral adiposity, which is most closely related to insulin resistance and metabolic alterations. Indeed, imaging (e.g., visceral adipose volume) and bioelectrical impedance analysis (e.g., whole-body fat mass) are more accurate measurements of body composition and are better predictors of MASLD^15^.

Here, we leverage these direct measurements of adiposity to identify novel genetic *loci* associated with MASLD. We identified and replicated 6 novel *loci* and generated two partitioned polygenic risk scores (PRS) based on the mechanism of genetic association with MASLD encompassing intra-hepatic lipoprotein retention. The two PRS suggest the presence of at least two distinct types of MASLD, one confined to the liver and one entwined in the systemic cardiometabolic syndrome.

## Results

### Visceral adipose tissue, whole-body fat mass and BMI are independent predictors of liver triglyceride content and inflammation

**Figure 1:**
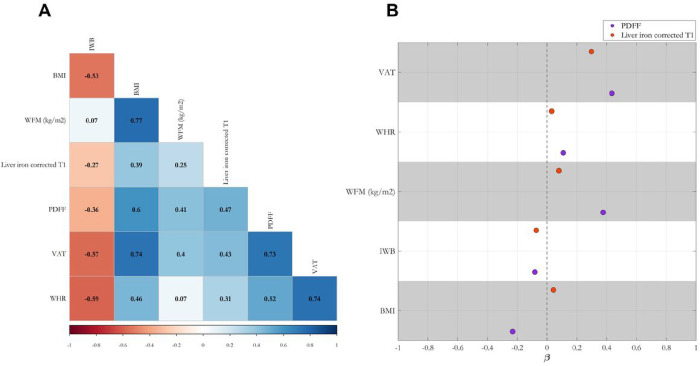
(A) Measures of adiposity are highly correlated with liver triglycerides and inflammation, and (B) among these measures, visceral adipose tissue (VAT), whole-body fat mass (WFM), and body mass index (BMI) are independent predictors of the liver outcomes (B). In (A), the phenotypic correlation between different measures of adiposity, liver triglyceride content measured by proton density fat fraction (PDFF), and inflammation measured by liver iron corrected T1 (cT1); pairwise Spearman’s correlation coefficients have been shown on the heatmap. All correlations had a Benjamini-Hochberg False Discovery Rate (FDR) < 0.05. (B) penalized Ridge regression analysis of different adiposity indices in predicting PDFF and liver iron corrected T1. Each dot represents standardized coefficients, and dashed line represents the lack of contribution of each trait to the liver outcomes. Both target variables were rank-based inverse normal transformed before the regression analysis. IWB: impedance of whole body, WHR: waist-to-hip ratio.

We started by examining the pairwise correlation among different measures of adiposity and a) liver triglyceride content measured by MRI-derived proton density fat fraction (PDFF), and b) liver inflammation/fibrosis measured by liver iron corrected T1 (cT1, Fig. 1A). The strongest correlation with liver outcomes was observed with visceral adipose tissue volume (VAT) followed by BMI, waist-to-hip ratio (WHR) and whole-body fat mass (WFM). As expected, there was a high correlation between PDFF and cT1. Due to the presence of high multicollinearity among the adiposity indices, we carried out a model selection approach using 3 penalized regression models (see Methods) to dissect the predictive contribution of these measures of adiposity to PDFF and cT1. The minimum mean standard error (MSE) on the validation set showed that the Ridge regression model outperformed LASSO and Elastic Net models. The standardized coefficients from the Ridge regression showed that VAT was the strongest independent predictor of PDFF and cT1 followed by WFM and BMI for PDFF (Fig. 1B). Interestingly, due to high collinearity between WFM and BMI, L2 regularization term resulted in a negative standardized coefficient for BMI in predicting PDFF. As opposed to the pairwise correlation, in the penalized regression analysis WHR and IWB had almost no independent predictive power in determining PDFF or cT1. Therefore, we used WFM, BMI and VAT for the following genetic association studies.

### Identification of 17 novel loci for liver triglyceride content and 9 for liver inflammation by the multi-adiposity-adjusted GWAS

**Figure 2:**
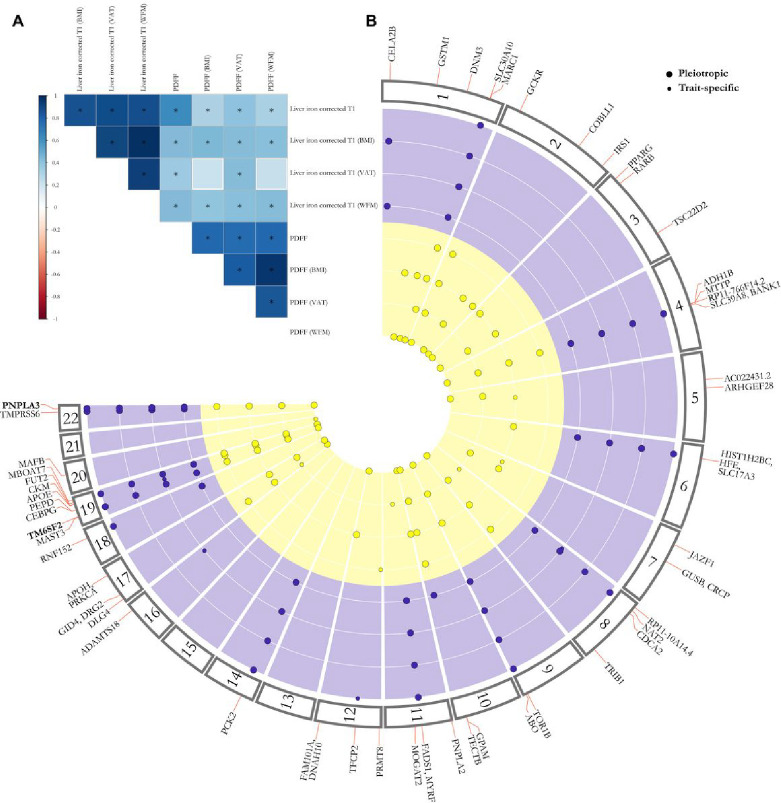
(A) BMI and whole-body fat mass (WFM) have the highest genetic correlation with liver triglycerides content (PDFF) and inflammation (cT1), and (B) 37 for liver triglycerides and 18 for liver inflammation independent *loci* were found by the multi-adiposity-adjustment GWAS. (A) Genetic correlation among different multi-adiposity-adjusted PDFF and liver iron corrected T1 was estimated using LD score regression analysis. The asterisks denote Benjamini-Hochberg False Discovery Rate (FDR) < 0.05. The colour bar represents the genetic correlation values. Detailed summary statistics for genetic correlations have been reported in Supplementary Table 2. (B) Circular Manhattan plot of PDFF and liver iron corrected T1 for different adiposity adjustments. Each dot represents an independent genetic locus. The yellow represents *loci*associated with liver PDFF and purple those associated with liver cT1. Large dots represent pleiotropic *loci,* namely *loci* where the association with either PDFF or liver cT1 was shared among two or more adiposity adjustments. Small dots show adiposity-trait specific associations. *Loc*in bold are shared among both traits irrespective of the adiposity adjustment. Only *loci* with a genome-wide significant p-value < 5E-8 calculated by whole-genome regression model (see methods) are shown. P-values were not corrected for multiple testing among 4 different models (unadjusted, adjusted for BMI, WFM and VAT). VAT: Visceral adipose tissue; WFM: Whole body fat mass (kg/m2).

To capitalize on the independent contribution of indices of adiposity to liver lipid content and inflammation, we performed three GWAS each of them adjusted for a specific index of adiposity (VAT, BMI, WFM) and one unadjusted, namely multi-adiposity-adjusted GWAS. All the GWAS were between 9,356,431 common genetic variants (MAF >0.01) and PDFF (N = 36,394) and cT1 (N = 30,481) in Europeans from the UK Biobank and adjusted for age, sex, agexsex, age^2^ and age^2^xsex, first 10 genomic principal components and array batch^16^. We estimated genetic correlation and heritability using linkage disequilibrium (LD) score regression analysis^17–19^. For PDFF, WFM adjustment explained the largest genetic variability followed by BMI and VAT explaining in the best-case scenario approximately 6% more heritability compared to the model unadjusted for any adiposity index (Supplementary Table 1). For cT1, all adiposity measurements yielded similar results to the unadjusted model. Intercepts from the LD score regression analysis did not show any sign of substantial confounding bias (Supplementary Table 1). These data suggest that while liver triglyceride content is dependent on adiposity, the presence of inflammation is less closely correlated to it. Furthermore, genetic correlations among different adiposity adjustments showed that BMI and WFM adjustments shared the largest overlap for both PDFF and cT1 (Fig. 2A, Supplementary Table 2), which is consistent with the epidemiological correlation.

To identify statistically independent genetic *loci* for each adiposity adjusted GWAS, we performed linkage disequilibrium (LD) clumping^20^ (r^2^ >0.01 in a window of 1 Mb), followed by conditional and joint multiple-SNP analysis (GCTA-COJO)^21^. Next, to identify independent genetic *loci* from all 4 adiposity adjustments we used pleiotropic analysis^22^, by examining linkage disequilibrium among independent lead variants from the 4 adiposity adjustments and kept the strongest independent association as the lead variant. In this context, pleiotropic analysis refers to genetic *loci* that are shared among more than two adiposity adjustments (Supplementary Table 3).

A total of 37 and 18 independent genetic *loci* for PDFF and cT1 reached the genome-wide significance level (P < 5E-8 not adjusted for the four different GWAS carried out, namely three adipo-adjustments and one unadjusted), respectively (Fig. 2B, Table 1). Of these 37 independent *loci* for PDFF, 2 *(PPARG* and *MAFB),* 2 *(ARHGEF28* and *JAZF1),* 5 *(MTTP, CDCA2, MOGAT2, DLG4* and *CEBPG)* and 3 *(PRMT8, MAST3* and *CKM)* had the strongest association when adjusting for WFM, BMI, VAT and no adiposity adjustment, respectively. Similarly, 3 *(RP11–10A14.4, ADAMTS18* and *PEPD)* and 1 *(TFCP2) loci* for cT1 showed the strongest association when adjusted by VAT and no adiposity adjustment, respectively (Supplementary Table 4).

To examine whether the newly identified genetic *loci* were previously reported, we searched NHGRI-EBI GWAS Catalog database^23^ in a window of 1 Mb around each lead variant. We found 17, and 9 novel genetic *loci* associating with PDFF and cT1, respectively (Tables 1 and 2, Supplementary Table 5). Among the new and the previously identified *loci,* 4 *(PNPLA3, TM6SF2, GPAM, HFE/SLC17A3)* were associated with both traits with at least one adiposity adjustment. However, only *PNPLA3* and *TM6SF2 loci* were associated with both traits at a genome-wide level irrespective of the adjustment (Fig. 2B).

### Identification of the putative causal loci associated with liver triglyceride content and inflammation

We next fine-mapped the independent genome-wide significant *loci* associated with adiposity-adjusted PDFF and cT1. Per-SNP heritability estimates calculated by PolyFun^24^ were used as prior causal probabilities in sum of single effects (SuSiE)^25^. Independent lead variants at *GSTM1, MARC1, GCKR, RARB, TSC22D2, ADH1B, CDCA2, TRIB1, TOR1B, PNPLA2, TM6SF2* and *APOE* for PDFF, and *SLC30A10, SLC39A8* and *PCK2* for cT1 had a posterior inclusion probability (PIP) >0.95, suggesting the GWAS lead variants at those *loci* are potentially the causal variants (Supplementary Table 6). This was followed by the lead variants at *HFE* (PIP = 0.78), *PRMT8* (PIP = 0.74), *APOH* (PIP = 0.93) and *TMPRSS6* (PIP = 0.73) *loci* with a less robust evidence of being the putative causal variants.

Interestingly, a missense variant on *ADH1B* (rs1229984) was in the first credible set with a PIP of 1 at *ADH1B, MTTP* and *RP11–766F14.2 loci,* suggesting that the observed effect from all three locimay derive from the same putative causal variant. Although SuSiE identified 2 credible sets at *MTTP* locus, the second credible set contained 49 variants with highest PIP of only 0.08. In fact, *ADH1B* rs1229984 and *MTTP* rs11937107 have a D’ of 1 in Europeans^26^. Similarly, fine mapping identified 3 credible sets at *MAST3* locus, but *TM6SF2* rs58542926 was the only variant in the first credible set with a PIP of 0.98. We observed a similar finding at *CKM* locus with 2 credible sets, with a large PIP (>0.99) for *APOE* rs429358 in the first credible set. Of note, an upstream gene variant near *CEBPA* had a PIP of 0.84 at *CEBPG* locus that recently was found associated with chronic ALT levels in a recent multi-ancestry GWAS^9^. For other *loci,* independent variants from GWAS were among the fine-mapped credible sets, but with a relatively low probability of being the putative causal variant at the respective *loci* (Supplementary Table 6). SuSiE failed to identify any credible set for *FAM101A* and *CELA2B loci* possibly due to a high purity filter (r^2^< 0.25).

To examine whether the set of independent variants could potentially perturb the gene expression patterns of nearby genes, we performed a Bayesian colocalization with gene expression quantitative trait *loci* (eQTL) summary statistics of 49 tissues from Genotype-Tissue Expression (GTEx) project (v.8) uniformly processed by eQTL Catalogue (Supplementary Table 7)^27–29^. We were able to colocalize 13 and 7 GWAS signals with at least one eQTL evidence for PDFF and cT1, respectively. Among these, *MBOAT7* (same gene, H4.PP = 0.99), *RP11–10A14. 4*(*TNKS,* H4.PP = 0.99), *MYRF* (*FADS3,* H4.PP = 0.93) *loci* were colocalized with an eQTL signal in the liver.

### Functional analyses of independent loci associated with liver triglyceride content and inflammation

Independent genetic *loci* for adiposity adjusted PDFF and cT1 were mapped to genes using multiple approaches (see Methods). We performed positional eQTL in a window of 50 kb, and chromatin interaction mapping using FUMA^30^. A gene-based association analysis of common variants was performed in MAGMA^31^ to find potential coding genes associating with PDFF or cT1. We further used the V2G score for each leading variant at each locus from Open Target Genetics, along with the set of genes found *via* colocalization and fine-mapping. To rank the potential genes at each locus, we summed the evidence for each approach (Supplementary Table 8). This approach allowed us to map 724 unique genes with at least one piece of evidence for PDFF, and 469 for cT1.

Out of 37 and 18 independent *loci* for PDFF and cT1, the majority (31 and 12) *loci* had the highest rank for the nearest genes, respectively (Supplementary Table 8). For the remaining *loci,* multiple candidate genes were found. Specifically, while 3 genes, *GID4, MYO15A* and *ATPAF2* had the highest rank at *GID4* locus, in a recent report^8^, this locus has been attributed to *SREBF1* with at least one significant eQTL association and chromatin interaction (FUMA), and a significant association at the gene level (MAGMA). At *FAM101A* locus, *CCDC92* had the highest rank. Whole-body knockout of this gene in a mouse model has been found to be protective against obesity and diabetes^32^. In addition, *SLC2A4,* an insulin regulated transporter of glucose, and *ACADVL,* an acyl-CoA dehydrogenase catalysing the first step of mitochondrial fatty acid beta-oxidation, had the highest rank at *DLG4* locus. *LETMD1* had also the largest rank at *TFCP2* locus. This gene plays a role in thermogenesis of brown adipose tissue, and mice lacking this gene were susceptible to diet-induced adiposity and impaired glucose disposal^33^.

To gain a deeper understanding of the biological implications of genome-wide significant *loci,* we conducted functional gene-set enrichment analysis using gene ontology (GO) biological processes, Reactome metabolic pathways, and ARCHS4 tissues. We focused on the mapped genes with the highest evidence rank at each locus (Supplementary Tables 9A and B). Mapped genes for PDFF were enriched in genes mostly expressed in liver and they were involved in triglyceride and lipid metabolism (Supplementary Table 9A and Supplementary Fig. 1A). Conversely, mapped genes for liver iron corrected T1 were enriched in metal ion metabolism and biosynthesis of blood groups (Supplementary Table 9B and Supplementary Fig. 1B).

## Most genetic variants were associated with both liver triglyceride content and inflammation

**Figure 3:**
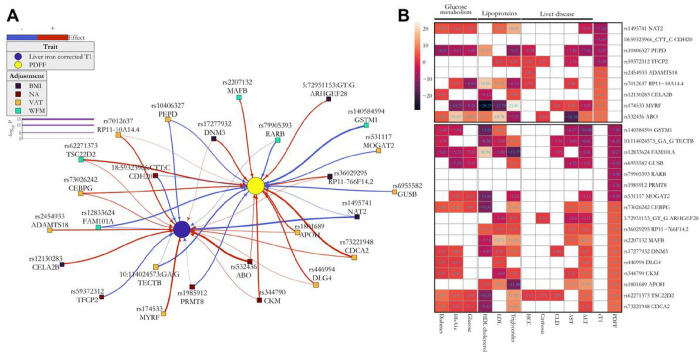
(A) A consistent reciprocal trait association between novel *loci* and liver triglycerides (PDFF, yellow dot) and inflammation (purple dot, cT1) and (B) novel genetic *loci* were associated with liver disease and metabolic traits. (A) Association was calculated by a whole-genome regression analysis and the colour of squares represent: no adjustment (red square) adjusted for BMI (black square), for whole-fat mass (WFM, green square) and visceral adipose tissue (VAT, orange square). The edge colours denote the direction of the association with the effect (risk) allele, and their thickness correspond to the strength of the association (−logl 0 P-value). (B) Heatmap of the Z-score of associations for the effect (risk) allele between novel genetic *loci* and liver or metabolic-related traits (columns) in n=397,780 UKBB participants after excluding individuals with available PDFF or liver iron corrected T1 (n=36,748). Upper and lower boxes correspond to liver iron corrected T1 and PDFF genetic *loci,* respectively. Full summary statistics have been reported in Supplementary Table 10. P-values were not corrected for multiple hypothesis testing. VAT: Visceral adipose tissue; WFM: Whole-body fat mass (kg/m2); cT1: liver iron corrected T1; PDFF: proton density fat fraction; CLD: chronic liver disease.

Given the causal relationship between liver triglyceride content and inflammation, we examined the association of the novel variants identified by PDFF with cT1 and *vice versa* (Fig. 3A). Interestingly, the majority of the variants were associated with both traits and directionally concordant (Fig. 3A). This is consistent with the notion that liver triglyceride content causes inflammation^34^. A total of 5 (29%) and 4 (44%) *loci* were associated with either PDFF or cT1, suggesting a specificity of the effect on lipid and inflammation pathways.

### Association between novel genetic loci and liver and metabolic traits

Next, we examined the association between these novel variants and indices of liver damage, fibrosis and liver disease (Fig. 3B and Supplementary Table 10). As expected, more than 80% of variants associated with PDFF also associated with ALT, while more than 50% associated with cT1 associated with AST. Among the variants shared for both traits, only 5 *(MYRF, TECTB, DNM3, FAM101A* and *TSC22D2 loci)* were associated with chronic liver disease, and 4 with cirrhosis *(TFCP2, ABO, ARHGEF28* and *TSC22D2 loci).* Strikingly, there was an enrichment of variants associated with hepatocellular carcinoma (HCC) in the variants deriving from cT1 (44%, one-sided Fisher’s exact test p-value = 0.035), but not in PDFF (6%). Most variants were associated with plasma lipoproteins and glucose metabolism traits, including diabetes (Fig. 3B and Supplementary Table 10).

### Polygenic risk scores of liver triglyceride content and inflammation explain approximately 6% of variation of these traits

We generated polygenic risk scores (PRS) for PDFF and cT1 using 1) all variants identified in the multi-adiposity-adjusted GWAS, 2) only previously known genetic variants, and 3) novel variants identified in the present study. We calculated goodness-of-fit of PRS using overall variance explained (% R^2^, Supplementary Table 11). Full PRS explained approximately 5.6% and 7% of the phenotypic variance for PDFF and cT1, respectively. As expected, this variance was mostly accounted for by previously known variants, where novel PRS conferred an improvement in prediction of less than 1 % of phenotypic variance for both traits (Supplementary Table 11).

### The association between six novel loci and liver triglyceride content was replicated in independent cohorts

**Figure 4:**
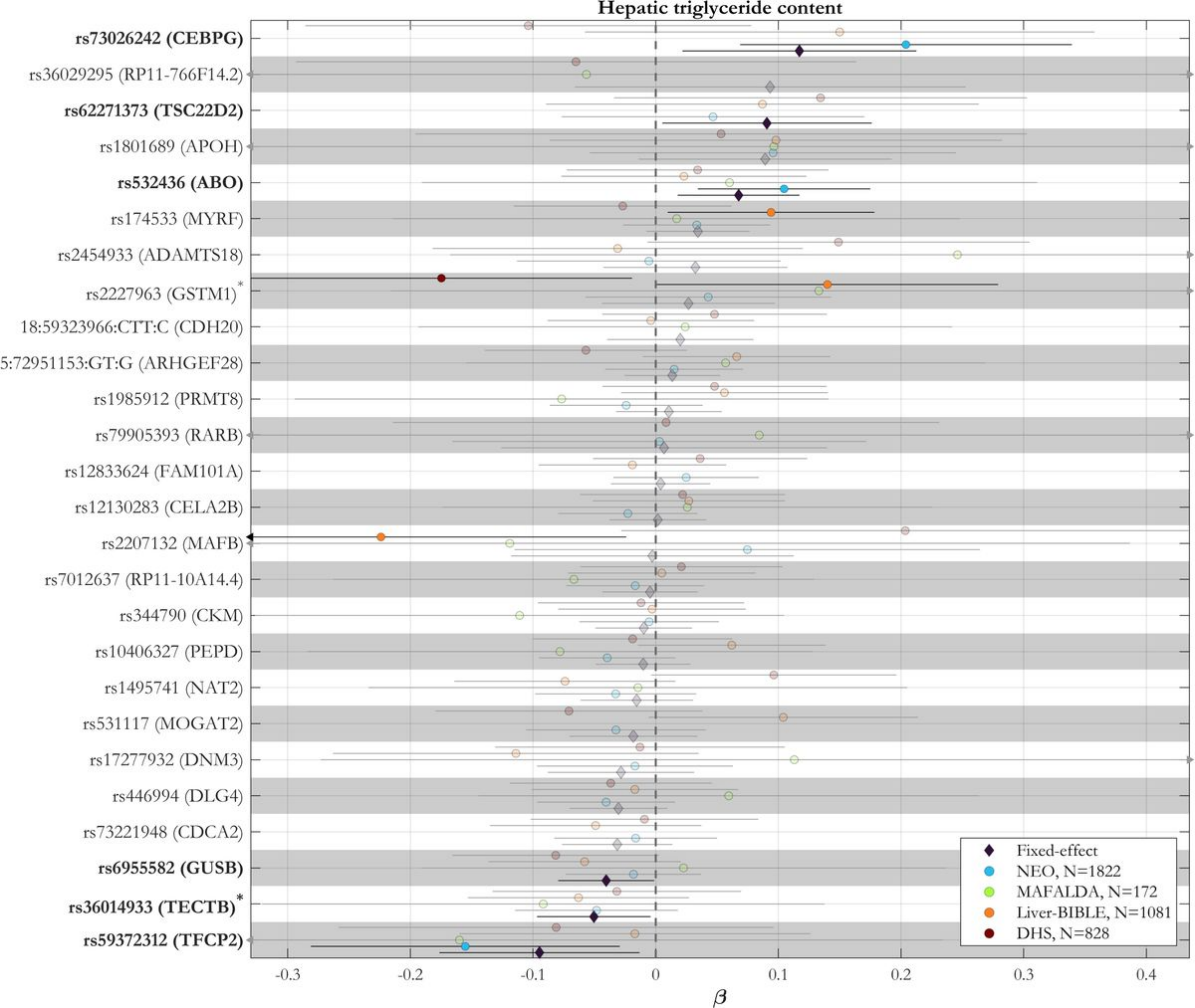
The association between 6 novel *loci* and hepatic triglyceride content was replicated in independent cohorts. Meta-analysis of the associations between independent novel genetic *loci* and hepatic triglyceride content in four independent European cohorts. Proxy variants were used for variants not available in the replication cohorts (r2 > 0.4 within a window of 1.5 Mb around each lead variant in the UK Biobank) as marked with an asterisk. Pooled effect estimates were calculated using inverse-variance-weighted fixed-effect meta-analysis. Genomic *loci* in bold are those with a P-value < 0.05 in the fixed-effect model. Full summary statistics have been reported in the Supplementary Table 12. P-values are two-sided and not adjusted for multiple testing.

Based on the strong genetic correlation between PDFF and cT1, to validate the novel SNPs, we meta-analysed the association between all the novel 26 variants and liver triglyceride content in 3,903 individuals of European ancestry from four independent cohorts (Fig. 4 and Supplementary Table 12). We were able to replicate the association between six of the novel *loci* (*CEBPG, TSC22D2, ABO, GUSB, TECTB, TFCP2*) and liver triglyceride content. The direction of the association in the replication cohort was consistent with the discovery cohort.

## Partitioned polygenic risk scores dissect a liver-specific from a cardiometabolic component of steatotic liver disease

Triglyceride secretion is a key mechanism regulating intracellular hepatocyte triglycerides homeostasis. Triglyceride secretion is mediated by very low-density lipoprotein (VLDL) secretion that in fasting conditions are proxied by circulating triglyceride levels. Variants in genes hampering incorporation of lipid and directly VLDL secretion, including *APOB, MTTP, TM6SF2* and *PNPLA3,* cause retention of liver triglycerides, cholesterol and other lipid species mirrored by lower circulating triglycerides and low-density lipoprotein cholesterol. Carriers of these variants have an increased risk of the entire spectrum of steatotic liver disease and at the same time lower risk for cardiovascular disease due to the lower circulating lipoproteins^35–37^. Moreover, *MTTP* inhibition is currently used as a strategy to reduce cardiovascular risk in genetically determined hypercholesterolemia.

**Figure 5:**
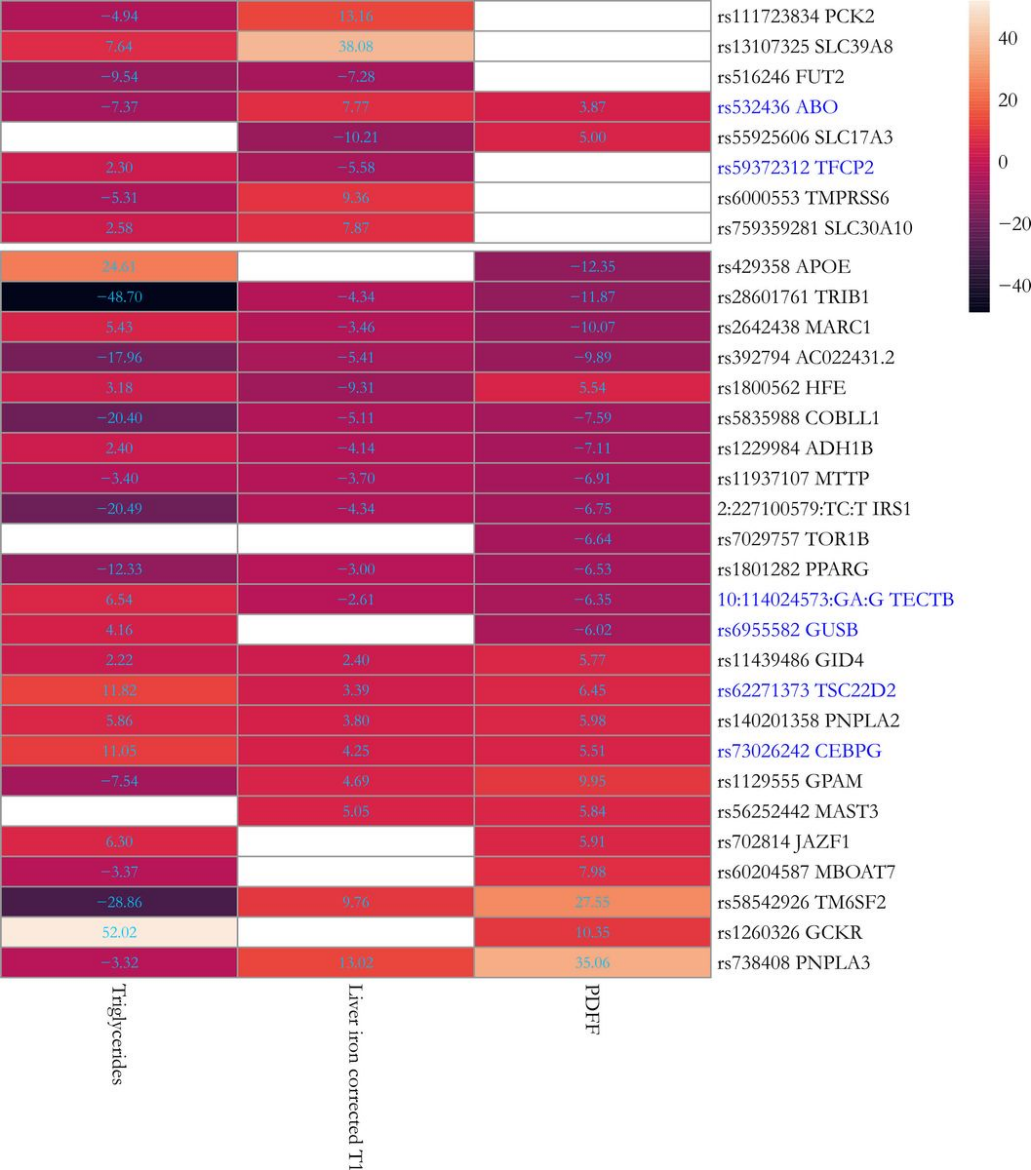
Association between 26 previously known and 6 novel replicated genetic *loci* and circulating triglycerides in the UK Biobank. The heatmap shows the Z-score of associations for the effect (risk) allele in Europeans (n=397,780) after excluding individuals with available PDFF or liver iron corrected T1 (n=36,748). Upper and lower boxes correspond to liver iron corrected T1 and PDFF genetic *loci,* respectively. Novel replicated genetic *loci* have been marked in blue. Full summary statistics have been reported in Supplementary Table 13. P-values were not corrected for multiple hypothesis testing.

Based on this fundamental mechanism, we clustered the newly replicated and the previously known variants in two groups, and generated two partitioned PRS of PDFF-circulating triglycerides: a) a cluster with discordant association between PDFF and circulating triglycerides (n = 10), where the primary cause of higher liver triglyceride content is likely to be retention and b) a cluster with concordant direction (n = 13), where the primary cause may be an increase in uptake and synthesis of energy substrates. Variants at three genetic *loci (SLC17A3, TORI B* and *MAST3)* did not associate with circulating triglycerides and were not included (Fig. 5 and Supplementary Table 13). While goodness-of-fit measures of clustered PDFF-circulating triglycerides showed a strong association for the two PRS, the explained variance of discordant PRS was comparatively higher than the concordant. This was expected, as discordant PRS is composed by *PNPLA3* and *TM6SF2* variants, the strongest genetic predictors of liver triglyceride content (Supplementary Table 11).

**Figure 6:**
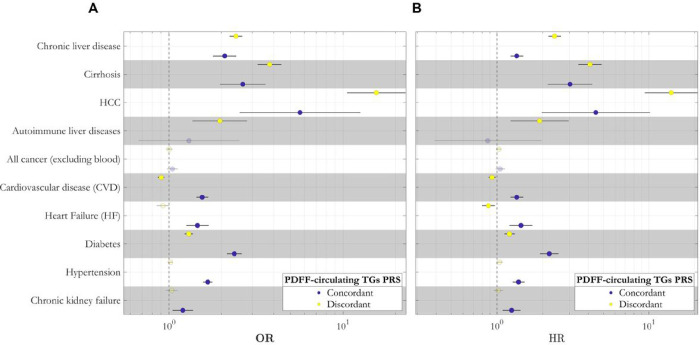
Polygenic risk scores (PRS) dissect a liver specific (discordant) and a cardiometabolic (concordant) subtypes of steatotic liver disease. The case-control (A) and prospective (B) association between 2 clustered PDFF-circulating TGs PRS and liver-related, cardiometabolic, and chronic kidney failure traits in the UK Biobank. Effect plot of the association between concordant and discordant PDFF-circulating TGs PRS with each disease was tested using either logistic (A) or Cox proportional hazard (B) regression analysis adjusted for BMI, age, sex, agexsex, age^2^ and age^2^xsex, first 10 genomic principal components and array batch. X-axis shows either the odds ratio (OR) or hazard ratio (HR). All association analyses have been performed after excluding individuals with available PDFF. Full summary statistics have been reported in Supplementary Table 15. P-values were not corrected for multiple hypothesis testing. TG: triglyceride; PDFF: proton density fat fraction; HCC: hepatocellular carcinoma; PRS: polygenic risk scores.

Discordant PRS was associated with an increased risk of the entire spectrum of steatotic liver diseases with the largest association being with hepatocellular carcinoma. The concordant PRS had an overall similar effect on the risk of chronic liver disease. However, the difference in effect size with the discordant PRS was decreasing with increasing severity of liver disease to cirrhosis and cancer (Fig. 6A, Supplementary Fig. 2, and Supplementary Tables 14 and 15). Interestingly, the discordant PRS, but not the concordant, was also associated with autoimmune liver disease.

Discordant PRS was associated with a decreased risk of cardiovascular disease. On the contrary, concordant PRS was associated with a substantial increased risk of cardiovascular disease and heart failure. When examining diabetes, both discordant and concordant PRS conferred an increased predisposition to this condition, suggesting that hepatic fat accumulation predisposes to diabetes irrespective of the underlying mechanism. Conversely, the larger effect size of the concordant PRS for diabetes despite the lower effect on liver triglyceride content would suggest that the association of diabetes in the concordant PRS is not mediated by liver damage. In the case of hypertension and chronic kidney failure, discordant PRS showed no association, whereas the concordant PRS increased the risk of both diseases. However, when we adjusted for hypertension, the association with chronic kidney failure was no longer significant while the other associations remained (Supplementary Table 15). Further adjustment of diabetes, total cholesterol and alcohol intake did not change the results (Supplementary Table 15). When examined the prospective risk conferred by the partitioned PRSs to develop liver and cardiometabolic disease we found virtually identical results (Fig. 6B, Supplementary Table 15).

Functional gene-set enrichment analysis of mapped genes for discordant and concordant PRS also revealed a distinct metabolic pattern. While gene sets of discordant PRS were mostly enriched in lipid and triglycerides homeostasis (Supplementary Table 9C, Supplementary Fig. 3), concordant PRS gene sets were enriched in insulin receptor signalling and glucose homeostasis pathways, overall consistent with an impact on stimulation of *de novo* lipogenesis (Supplementary Table 9D, Supplementary Fig. 3).

### mRNA expression of lod from the liver specific PRS is more abundant in the liver

**Figure 7:**
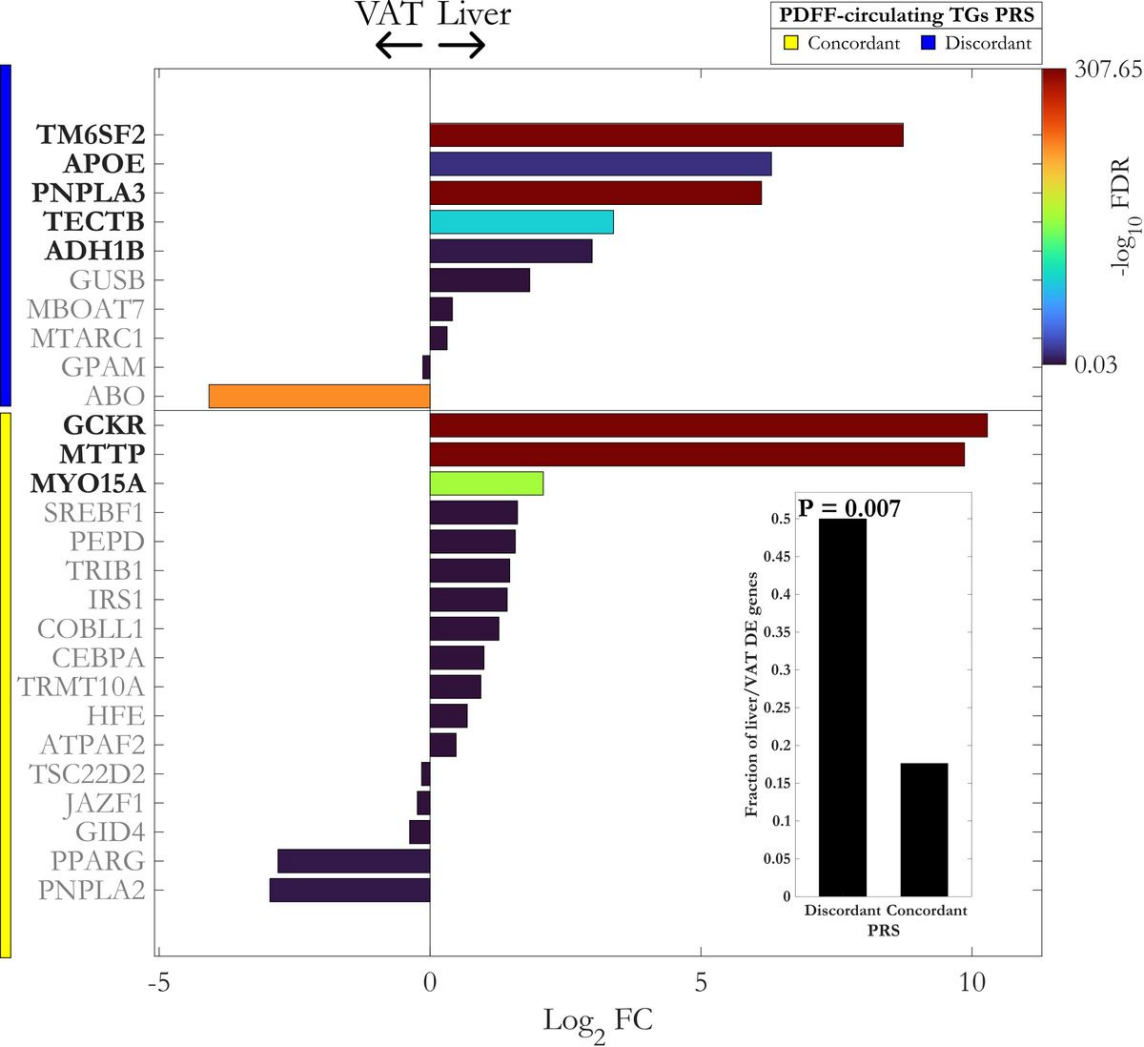
mRNA expression of *loci* from the liver specific (discordant) polygenic risk score is more abundant in the liver compared to the visceral adipose tissue (VAT). Differential expression analysis of paired liver and VAT bulk RNA-Seq data for mapped gene sets of concordant and discordant PRS. The lower right bar plot shows the fraction of upregulated differentially expressed (DE) genes in the liver compared to VAT. The enrichment of PRS clusters with upregulated DE genes in the liver was calculated using one-sided Fisher’s exact test. VAT: visceral adipose tissue; FC: fold change; FDR: false discovery rate.

We further examined the mRNA expression of mapped-genes within concordant and discordant PRS using paired bulk RNA-Seq of liver (n = 244) and visceral adipose tissue (VAT, n = 261) from participants with obesity from the MAFALDA cohort. Interestingly, mapped genes of discordant, but not the concordant PRS, showed a significant overlap with upregulated differentially expressed genes in the liver (one-sided Fisher’s exact test p-value= 0.007, Fig. 7). Given the tight interplay between VAT and liver in the SLD, this finding suggests a liver specific nature of discordant PRS compared to its metabolic counterpart, concordant PRS.

## Discussion

The main findings of this study are: a) the identification of novel *loci* associated with steatotic liver disease and b) the identification of two distinct types of MASLD, namely a liver specific and a systemic cardiometabolic.

BMI, as a proxy of adiposity, amplifies the genetic predisposition to SLD given by common variants^14,38^. However, BMI does not consider body fat distribution and body composition. Based on this consideration, to identify novel genetic *loci* associated with MASLD, we compared a range of instrumental and anthropometric measurements of adiposity. We found that visceral adipose tissue volume, whole-body fat mass and BMI were the best independent predictors of liver triglyceride content and inflammation measured by PDFF and cT1, respectively.

Next, we performed multi-adiposity-adjusted GWAS on PDFF and iron corrected T1 content, as a measure of liver triglyceride content and inflammation. We identified a total of 17 novel genetic *loci* for liver triglyceride content and 9 for liver inflammation and replicated 6 of these *loci* in four independent cohorts. Among the previously known, we found *loci* associated with lipid droplet homeostasis in hepatocytes *(PNPLA,3 TM6SF2, MBOAT7, MARC1, GPAM* and *APOE).*

The heritability of liver triglyceride content was influenced by the multi-adiposity adjustment explaining in the best-case scenario approximately 6% more heritability compared to the unadjusted. However, for inflammation this was not the case, suggesting that heritability of inflammation is not directly influenced by adiposity. Liver triglyceride accumulation is causally associated with liver inflammation^34^. Consistently, approximately 80% of the genetic *loci* were associated with both liver triglyceride content and inflammation.

When we examined the association among liver traits and the novel variants, we found that 80% of those associated with liver triglyceride content were associated with alanine transaminase (ALT), a clinical biomarker of liver triglyceride accumulation and 50% of those associated with liver cT1 were associated with aspartate transaminase (AST), a marker of liver damage^39^. Interestingly, approximately 50% of the variants associated with cT1 were also associated with hepatocellular carcinoma (HCC) while only 6% of those associated with liver triglyceride content were associated with this cancer. This is consistent with cT1 measuring inflammation and liver damage, which may indicate a more advance disease stage, as opposed to PDFF measuring purely triglyceride content.

Intrahepatocyte triglyceride homeostasis is governed by three fundamental pathways: triglyceride synthesis, lipoprotein secretion, and energy substrate utilization. While all cells in the body are capable of synthesizing and utilizing triglycerides, lipoprotein secretion during fasting is specific to the hepatocyte. Lipoprotein secretion consists of the incorporation of triglycerides into very low-density lipoproteins as a getaway for partitioning lipids to the adipose/muscle tissues and, in fasting conditions, it is highly correlated with circulating triglycerides.

Hindering lipoprotein secretion causes liver triglyceride accumulation by retention. Indeed, rare loss of function mutations in *APOB* segregate in families with liver steatosis and hepatocellular carcinoma and are enriched in case studies with this cancer^40^. Moreover, loss of function variants in *TM6SF2* and *PNPLA3,* the strongest common genetic predictors of SLD, cause liver triglycerides retention by reducing lipoprotein secretion^41,42^. Counter-intuitively, carriers of these variants have lower risk for cardiovascular disease due to the lower circulating lipoproteins^35,43^.

Therefore, we decided to generate two partitioned PRS: one composed by variants in which the association between liver triglyceride content and circulating triglycerides were discordant and one in which they were concordant. “Partitioned” or “process-specific” polygenic scores define specific pathways elucidating disease pathogenesis and identifying opportunities for drug target identification. These partitioned scores may also capture the specific signature driving the individual progression from health to disease, hence providing a framework for tailored therapeutic interventions^44^.

The discordant PRS was composed by genetic variants primarily causing hepatic triglyceride retention, whereas the concordant PRS by variants not affecting liver secretion but, allegedly, the other two pathways, namely triglyceride synthesis and utilization. Moreover, the two PRS confer primarily a large risk for the entire spectrum of MASLD where the severity of liver disease was accompanied by a larger genetic risk. However, the discordant confers a substantially larger effect size as compared to the concordant PRS.

To the best of our knowledge, the concordant is the first PRS predicting the entire spectrum of cardiometabolic disease, namely MASLD, diabetes, heart failure, and cardiovascular disease. On the contrary, the discordant PRS is liver specific and associates with a more aggressive liver disease mirrored by protection from cardiovascular disease due to lipoprotein retention, despite a marginal increase in the risk of diabetes. The liver specificity of the discordant PRS score is further supported by a higher mRNA expression of genes composing this score in liver versus visceral adipose paired biopsies from individuals with obesity.

**Figure 8:**
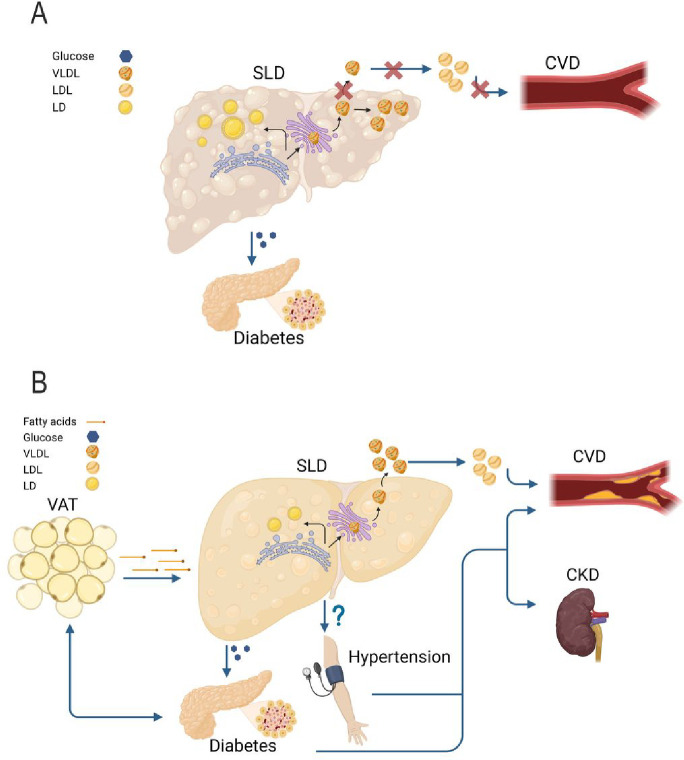
Putative model of the two different types of MASLD. A) In the liver specific MASLD, the primary increase in the liver triglyceride content is due to the hepatic retention of very low-density lipoproteins (VLDL). This retention is causally related to liver inflammation, fibrosis, and hepatocellular carcinoma. In this type of MASLD, the higher risk of diabetes is due to the degree of liver fibrosis, while the lower risk of cardiovascular disease (CVD) to lipoprotein retention. B) In the cardiometabolic MASLD, the liver is entwined in the crosstalk among metabolic organs. In this type of MASLD, a dysfunctional visceral adipose tissue may increase the diabetes risk and may release free fatty acids that are incorporated into triglycerides in the hepatocytes causing liver steatosis. In turn, liver steatosis causes an overproduction of VLDL with a subsequent increase in circulating low-density lipoproteins (LDL) resulting in a higher risk of CVD. Additionally, the cardiometabolic MASLD associates with an increased blood pressure resulting in kidney failure and further increasing the CVD risk. This figure was created with BioRender.com. CKD: chronic kidney disease (failure); VAT: visceral adipose tissue; LD: lipid droplets.

Our data suggest the presence of at least two distinct types of MASLD with specific disease-causing molecular mechanisms: one more aggressive, specific for the liver, and one associated with milder risk of liver events, but systemic and entwined with the cardiometabolic syndrome (Fig. 8). Understanding the molecular mechanisms underlying these components may allow to find effective treatments for MASLD and the cardiometabolic syndrome. Clinically, these entities reflect the presence of individuals fast progressing into the later stages of MASLD and those with a persistent, but slow progressing MASLD associated with the entire metabolic cardiometabolic syndrome. The presence of these MASLD subtypes may account for the disease heterogeneity and may contribute to explain why several drugs have failed in clinical trials to treat MASLD.

Currently mendelian randomization studies are done by selecting variants associated with a trait and using them to explain the causal relationship with a different trait. In this study, the PRS had opposite effects on cardiovascular risk indicating that if we had pooled the variants all together, we may have nullified the association. Therefore, our findings support the notion that the development of partitioned PRS constructed by integrating genetic variants into physiological pathways contributing to the trait phenotype, as compared to overall PRS based on the association between variants and a trait, may allow to better clarify the heterogeneity of disease pathogenesis at the individual level. Ultimately, this will lead to precision medicine improving outcome prediction and therapy.

A strength of this study is that the partitioning of the PRS was a hypothesis driven approach based on a solid knowledge of intracellular lipid homeostasis. While the finding on cardiovascular disease may be expected, although the adjustment for circulating total cholesterol did not changed results, the association with heart failure, hypertension and diabetes was not granted. An unsupervised clustering approach derived from several phenome-wide associations may be used for PRS partitioning. However, this approach may be considered somewhat a self-fulfilling prophecy because it uses traits to generate clusters that are subsequently used to predict diseases deriving from the same traits used in the clustering.

In conclusion, by leveraging human genetics and multi-adiposity adjustment we identified 6 novel *loci* associated with SLD and two distinct types of steatotic liver disease, namely one that is liver specific and confers risk of a more aggressive liver disease and another that is milder and entwined with the full spectrum of cardiometabolic syndrome.

## Methods

### UK Biobank.

The UK Biobank study has recruited over 500,000 participants aged between 40 and 69 years across the UK between 2006 and 2010, with extensive phenotypic and genetic data^45,46^. The UK Biobank received ethical approval from the National Research Ethics Service Committee North West Multi-Centre Haydock (reference 16/NW/0274). Data used in this study were obtained under application number 37142. European ancestry was defined as described before^12^ by removing outliers using genomic principal components. Additionally, subjects were excluded if they fall into any of these categories: 1) more than 10 putative 3rd degree relatives, 2) a mismatch between self-reported and genetically inferred sex, 3) putative sex chromosome aneuploidy, 4) heterozygosity and missingness outliers, and 5) withdrawn consent^45,46^.

### Genotypes and imputation.

UK Biobank participants were genotyped using 2 highly similar (> 95% overlap) genotyping arrays, which were then imputed centrally by the UK Biobank based on the 1000 Genomes Phase 3, UK 10K haplotype, and Haplotype Reference Consortium reference panels. Starting from approximately 97 million variants, we only kept 9,356,431 variants with a minor allele frequency (MAF) > 1 %, imputation quality (INFO) score > 0.8, and Hardy–Weinberg equilibrium P > 1E-10^12–46^.

### Definition of traits.

We used adiposity measures directly provided by UK Biobank, including visceral adipose tissue volume (VAT, data-field 22407), whole body fat mass (WFM, data-field 23100), impedance of whole body (IWB, data-field 23106). Waist-to-hip ratio (WHR) was calculated by dividing waist to hip circumference. MRI-derived proton density fat fraction (PDFF) and liver iron corrected T1 (cT1) were provided directly by UK Biobank (data-fields 40061 and 40062). The details on liver MRI protocols can be found elsewhere^47^. Briefly, individuals were scanned using a Siemens 1.5T Magnetom Aera. Two sequences were then used for data acquisition, a multiecho-spoiled gradient-echo and a modified look locker inversion sequence (ShMOLLI) for PDFF and cT1, respectively^47^. The definition of binary traits can be found in Supplementary Table 16.

### Phenotypic prediction models.

To address the multicollinearity between different measures of adiposity and to verify their contribution in predicting PDFF and cT1 values, we fit penalized linear regression models and carried out a model selection in a 10-fold nested cross-validation (CV) using Least Absolute Shrinkage and Selection Operator (LASSO), Ridge and Elastic Net. LASSO penalizes the regression model using the L1-norm, effectively reducing the influence of non-contributing features to zero. On the other hand, Ridge regression utilizes the L2-norm, allowing it to shrink regression coefficients toward zero. Elastic Net combines elements of both LASSO and Ridge by incorporating both L1 and L2 penalties through a mixing parameter *a*.

To conduct the CV process, the dataset was initially divided into training (80%) and test (20%) sets. Within the training set, the outer CV assessed the performance of each model, while the inner CV was utilized for hyperparameter tuning. This tuning was accomplished by minimizing the mean squared error (MSE) across a grid of *a* and shrinkage values in each fold of the outer CV. The best performed model with the lowest MSE, was then trained on the entire training set within a 10-fold CV framework. Subsequently, its performance was evaluated using the remaining test set. Finally, the model with the optimal set of hyperparameters, determined in the previous step, was fitted to the entire dataset for final evaluation^48^. Adiposity indices were standardized before the training, while PDFF and cT1 values were rank-based inverse normal transformed. All models were adjusted for age, sex, age^2^, agexsex, age^2^xsex. All analyses were performed in MATLAB (Mathworks) R2023a.

### Genome-wide association analysis.

The association between 9 million imputed common variants and PDFF or cT1 under different adiposity adjustments under an additive genetic model was performed using whole-genome regression model as implemented in REGENIE (v3.2.8)^16^. The analysis was adjusted for age at MRI, sex, age^2^, agexsex, age^2^xsex, the first 10 PCs of ancestry, genotyping array and adiposity index, where adiposity index was VAT, WFM, BMI or no adiposity adjustments.

Similarly, we tested the association between independent lead variants from multi adiposity adjusted GWAS of PDFF and cT1, and other binary or continuous metabolic traits using either a logistic or linear whole-genome regression model in REGENIE, and adjusted for the same set of covariates including consistent adiposity adjustments. Individuals with an available PDFF or cT1 measurements were excluded prior to the association analysis. In cases where the trait was measured at baseline, we used waist-to-hip ratio (WHR) instead of VAT adjustment, since the latter was not available at the baseline. To fit the whole-genome regression model in step 1 of REGENIE, a subset of directly genotyped common variants (MAF >1%) was used. After excluding variants on long-range linkage disequilibrium (LD) and major histocompatibility complex regions, variants with a missingness < 0.01, and with Hardy-Weinberg equilibrium P> 1E-15 were retained. Finally, 146,833 markers left following an LD pruning with a window of 500,000 base pairs and pairwise r2< 0.1^20^. Continuous traits were rank-based inverse normal transformed before the analyses.

### Identifying independent variants.

We first performed LD clumping (PLINK v1.90b6.26 parameters: --clump-p1 5e-8 –clump-r2 0.01 –clump-kb 1000, after excluding individuals with third-degree or closer relatives^20,46^) to identify approximately independent *loci.* Next, to detect statistically independent variants, we conducted approximate step-wise model selection in conditional and joint multiple-SNP analysis implemented in Genome-wide Complex Trait Analysis (GCTA-COJO^21^, v.1.94.0), with an LD window of 10 Mb and using 50,000 randomly selected unrelated Europeans from the UK Biobank for in-sample LD structure, as described before^12^.

### Estimating heritability and genetic correlations.

SNP heritability and confounding bias were estimated with LD score regression analysis (LDSC, https://github.com/bulik/ldsc/)^17^ using the baseline LD model (version 2.2; https://data.broadinstitute.org/alkesgroup/LDSCORE/), containing 97 annotations, including functional annotations and MAF-/LD-dependent architectures^19^. Similarly, pairwise genetic correlations were calculated using LDSC analysis^49^ after excluding variants in major histocompatibility complex (MHC) region (chromosome 6: 25–34 Mb) due to the complex LD structure. Trait pairs with a Benjamini-Hochberg False Discovery Rate (FDR) < 0.05 were considered to have a significant genetic correlation. In all analyses, LDSC parameter chisq-max to an arbitrary large number (99999) to keep large-effect associations.

### Pleiotropy analysis.

We evaluated whether the independent genome-wide significant loci, adjusted for different adiposity measures, were specific to each adiposity measure, common between PDFF and cT1 GWAS, or shared across both. Therefore, if two independent lead variants within 1 Mb of each other were in LD (r^2^ > 0.2), they were assigned the same locus id (Supplementary Table 3). Circular Manhattan plots were visualized using Circos^50^.

### Functionally-informed fine-mapping.

Functionally-informed genetic fine-mapping was performed using PolyFun and Sum of Single Effects (SuSiE, v0.11.92)^24,25^. PolyFun was used to estimate per-SNP heritability using L2-regularized extension of stratified LD score regression (S-LDSC) and baseline LD model v2.2 containing 187 annotations^17–19,24,51^. The estimated per-SNP heritabilities were used as prior causal probabilities in SuSiE with a maximum of 10 causal variants in each region. The subset of 337,000 unrelated white-British individuals from UK Biobank were used for in-sample LD structure. After excluding MHC region on chromosome 6, fine-mapping per each locus was performed in a window of 1.5 Mb around the lead genetic variants (P< 5E-8).

### Colocalization.

Colocalization was performed between independent genetic *loci* identified by COJO-GCTA, and summary statistics of gene expression quantitative trait loci (eQTL) of 49 tissues in GTEx (v8) from eQTL catalogue release 4^27,28^. The coordinates of GWAS summary statistics were first converted from Build 37 to 38 using liftOver function of rtracklayer R package (v.1.54.0)^52^. We performed colocalization using COLOC-SuSiE assuming the presence of multiple causal variants (coloc R package^29,53^, v.5.1.0) with default priors, and considered variants in a window of 1.5 Mb around index variant at each locus. We considered only genes with at least one significant variant (FDR P< 0.1, eGenes), and performed the colocalization in a window of 1.5 Mb around each eGene. If SuSiE did not converge after 1000 iterations, conventional (single causal variant) coloc was used. Finally, a H4 posterior probability (PP) >0.8 was considered as strong evidence that both traits share the same causal variant.

### Variant annotation.

Independent genome-wide significant and fine-mapped variants were annotated using Ensembl Variant Effect Predictor (VEP) accessed from REST API (https://rest.ensembl.org/)^54^.

### Gene mapping and functional enrichment analysis.

To map and prioritize potential candidate genes for independent genetic *loci,* we employed multiple approaches: 1) SNP2GENE module of FUMA v1.5.4^30^ was used for positional mapping of lead variants to genes with a maximum distance of 50 kb, 2) eQTL mapping using FUMA by considering only genes with at least 1 significant eQTL association (FDR < 0.05), 3) 3D chromatin interaction mapping using FUMA by considering only significant interactions (FDR< 1E-6) within 250 and 500 bp upstream and downstream of TSS, respectively, 4) Multi -marker Analysis of GenoMic Annotation (MAGMA, v1.08)^31^ analysis implemented in FUMA was carried out to perform genome-wide gene association analysis using 19,535 curated protein coding genes. Only genes with a Bonferroni threshold below 0.05/19,535 = 2.56E-6 were kept for gene mapping. Variants within MHC region were excluded prior to the analysis. 5) Colocalized genes from colocalization analysis with at least one tissue and an H4 posterior probability (PP) >0.8, 6) Nearest gene(s) to the fine-mapped variants with the maximum PP per each locus, and 7) Gene with the highest overall V2G score at each locus based on Open Targets Genetics^55^. Finally, to prioritize the mapped genes, we calculated an unweighted ranking score by summing over the evidence from the above-mentioned approaches.

By using the set of genes with the maximum ranking score at each locus, we performed functional gene-set enrichment analysis using Enrichr tool^56^ against, ARCHS4 tissues^57^, Reactome biological pathways^58^ and Gene Ontology (GO) Biological Processes. Significant terms with a Benjamini-Hochberg FDR corrected P-value< 0.05 per each database, were reported. For visualization, both adjusted P-values and Enrichr combined scores (−log(P)×odds ratio) were used.

### Replication cohorts.

#### NEO.

The Netherlands Epidemiology of Obesity Study (NEO) is a population-based cohort study in men and women aged 45 to 65 years, with oversampling of individuals having BMI over 27 kg/m^2^ from Leiden and surrounding areas in the Netherlands. At baseline, 6,671 participants were included and around 35% of the NEO participants were randomly selected to undergo hepatic triglyceride content (HTGC) measurements by MRS. Genotyping was performed using Illumina HumanCoreExome-24 BeadChip and imputed to TOPMed reference genome panel^59^. In the present work, a total of 1,822 individuals of European ancestry with an available HTGC were used.

#### Liver-BIBLE.

The Liver-BIBLE-2022 cohort comprises 1,144 healthy middle aged individuals (40–65 years) with metabolic dysfunction (at least three criteria for metabolic syndrome among BMI ≥ 35 Kg/m^2^, arterial hypertension ≥ 135/80 mmHg or therapy, fasting glucose ≥ 100 mg/dl or diabetes, low HDL< 45/55 mg/dl in M/F and high triglycerides ≥ 150 mg/dl) who presented for blood donation from June 2019 to February 2021 at the Transfusion Medicine unit of Fondazione IRCCS Ca’ Granda Hospital (Milan, Italy)^60^. Hepatic fat content was estimated non-invasively by controlled attenuation parameter (CAP) with FibroScan^®^ device (Echosens, Paris, France). Genotyping was performed by Illumina GlobalScreeningArray (GSA)-24 v3.0 plus Multidisease Array (Illumina, San Diego, CA), and further imputed to TOPMed reference genome panel^61^. At the time of analysis, genomic data passing quality control with an available CAP measure were available for 1,081 patients of European ancestry.

#### MAFALDA.

The “Molecular Architecture of FAtty Liver Disease in patients with obesity undergoing bAriatric surgery (MAFALDA)” study started in May 2020 and ended in April 2022. It comprises a total of 468 consecutive participants with morbid obesity (BMI ≥ 35 kg/m^2^) that underwent bariatric surgery at Campus Bio-Medico University of Rome, Italy in whom SLD diagnosis was assessed by liver histology or vibration-controlled transient elastography including CAP measurement with FibroScan^®^ (Echosens, Paris, France)^62^. Liver fat content estimated by CAP was available for 172 individuals. Genotyping was performed in the same manner as that of the Liver-BIBLE cohort.

#### Dallas Heart Study.

In this study only 828 European Americans from the Dallas Heart Study (DHS-1) were used. The DHS is a population-based sample study of the Dallas County, Texas, USA, where liver triglyceride content was measured by magnetic spectroscopy. Details of this study can be found elsewhere^13^.

#### Meta-analysis.

The association between novel independent *loci* for PDFF and cT1 and MRS liver fat (Dallas Heart Study and NEO studies) or CAP measurement (MAFALDA and Liver BIBLE) was performed using a linear regression analysis adjusted for age, sex, age^2^, agexsex, age^2^xsex, and BMI after a rank-based inverse normal transformation of the response. An inverse-variance meta-analysis was then performed with fixed-effect model using the meta R package (v6.5.0). For genetic variants not available in either of replication cohorts, a proxy variant was used instead: variants in LD (R^2^ > 0.4) with the lead variant in the UK Biobank within a window of 1.5 Mbp. If no such variant was found in the UK Biobank, LDproxy tool with Europeans from 1000 G project was used instead^26^. In case of multiple proxy variants, the one with the highest LD and functional consequence was selected.

#### RNA-seq analysis.

Total RNA for 264 paired liver and visceral adipose tissue samples was isolated using miRNeasy Advanced Mini kit (Qiagen, Hulsterweg, Germany). RNA sequencing and library preparation was performed in a paired-end 150 bp mode using the Illumina NovaSeq PE150 (Novogene, China). Reads were aligned to GRCh38 reference genome by STAR^63^ (v2.7.10a) after quality check (FastQC software, Babraham Bioinformatics, Cambridge, UK) and trimming of low-quality reads and potential contaminating adapters by Trimmomatic^64^ (v0.39). Gene-level read counts were quantified by RSEM^65^ (v1.3.3) tool against the Ensembl (release 107). Samples with insufficient mapping specificity (uniquely to total mapped reads < 0.7) were excluded before the analysis. Finally, a paired differential expression analysis of 261 VAT and 244 liver samples was carried out using DESeq2^66^ (v.1.38.3), while adjusting for RNA Integrity Number (RIN), individual ID, and 5 surrogate variables detected by surrogate variable analysis^67^.

#### Follow-up analysis.

The longitudinal association of PRS with the occurrence of the outcomes was tested through Cox proportional hazard regression and expressed as hazard ratios with 95% confidence intervals. The median follow-up was 14.5 y, and individuals with any of diagnoses at the baseline were excluded prior to the analysis (Supplementary Table 16). The proportional hazard assumption was checked through the consideration of Schoenfeld residuals, and no violations were detected. Prospective associations were performed in R v4.0.2 (R Foundation for Statistical Computing, Vienna, Austria).

## Figures and Tables

**Table 1:** Genome-wide significant loci for multi-adiposity-adjusted PDFF and liver iron corrected T1 in the UK Biobank.

Trait	CHR	POS	Variant ID	Consequence	A1	A2	A1Freq	Beta	SE	p	Locus	MAGMA
PDFF (WFM)	**1**	**110232983**	**rs140584594**	**missense_variant**	**A**	**G**	**0.267**	**−0.049**	**0.007**	**1.42E-13**	**GSTM1**	
PDFF (BMI)	**1**	**172323134**	**rs17277932**	**intron_variant**	**A**	**G**	**0.145**	**0.049**	**0.008**	**5.91E-09**	**DNM3**	
PDFF (VAT)	1	220970028	rs2642438	missense_variant	A	G	0.296	−0.061	0.006	7.89E-24	MARC1	5.91E-14
PDFF (BMI)	2	27730940	rs1260326	missense_variant	T	C	0.392	0.063	0.006	4.23E-25	GCKR	4.51E-14
PDFF (WFM)	2	165501927	rs5835988	intergenic_variant	TG	T	0.408	−0.046	0.006	3.25E-14	COBLL1	3.58E-11
PDFF (WFM)	2	227100579	2:227100579_TC_T	intergenic_variant	TC	T	0.348	−0.042	0.006	1.63E-11	IRS1	
PDFF (WFM)	3	12393125	rs1801282	missense_variant	G	C	0.122	−0.059	0.009	6.61E-11	PPARG	
PDFF (WFM)	**3**	**25484121**	**rs79905393**	**intron_variant**	**A**	**G**	**0.036**	**−0.094**	**0.016**	**2.86E-09**	**RARB**	
PDFF (WFM)	**3**	**150066540**	**rs62271373**	**regulatory_region_variant**	**A**	**T**	**0.060**	**0.082**	**0.013**	**1.02E-10**	**TSC22D2**	
PDFF	4	100239319	rs1229984	missense_variant	T	C	0.024	−0.165	0.023	1.26E-12	ADH1B	
PDFF (VAT)	4	100503761	rs11937107	intron_variant	T	C	0.252	−0.044	0.006	4.87E-12	MTTP	8.20E-12
PDFF (BMI)	**4**	**100562374**	**rs36029295**	**intron_variant**	**GA**	**G**	**0.039**	**0.085**	**0.015**	**2.68E-08**	**RP11-766F14.2**	
PDFF (VAT)	5	55808342	rs392794	intron_variant	C	T	0.254	−0.063	0.006	5.09E-23	AC022431.2	6.83E-13
PDFF (BMI)	**5**	**72951153**	**5:72951153_GT_G**	**intron_variant**	**G**	**GT**	**0.462**	**0.033**	**0.006**	**3.45E-08**	**ARHGEF28**	
PDFF (BMI)	6	26093141	rs1800562	missense_variant	A	G	0.077	0.062	0.011	3.27E-08	HFE	
PDFF (BMI)	7	28172732	rs702814	intron_variant	C	T	0.492	0.035	0.006	3.73E-09	JAZF1	
PDFF (VAT)	**7**	**65431686**	**rs6955582**	**intron_variant**	**A**	**G**	**0.451**	**−0.034**	**0.006**	**1.72E-09**	**GUSB**	**1.03E-08**
PDFF (VAT)	**8**	**25464670**	**rs73221948**	**intergenic_variant**	**T**	**G**	**0.294**	**0.046**	**0.006**	**3.05E-13**	**CDCA2**	
PDFF (BMI)	8	126500031	rs28601761	intron_variant	G	C	0.420	−0.072	0.006	1.63E-32	TRIB1	4.72E-07
PDFF (VAT)	9	132566666	rs7029757	non_coding_transcript_exon_variant	A	G	0.096	−0.063	0.009	3.15E-11	TOR1B	
PDFF (VAT)	10	113910721	rs1129555	3_prime_UTR_variant	A	G	0.273	0.062	0.006	2.52E-23	GPAM	4.04E-14
PDFF (VAT)	**10**	**114024573**	**10:114024573_GA_G**	**intergenic_variant**	**G**	**GA**	**0.286**	**−0.039**	**0.006**	**2.24E-10**	**TECTB**	**4.90E-09**
PDFF (WFM)	11	823586	rs140201358	missense_variant	G	C	0.015	0.147	0.025	2.35E-09	PNPLA2	
PDFF (VAT)	**11**	**75456134**	**rs531117**	**downstream_gene_variant**	**T**	**C**	**0.157**	**−0.042**	**0.008**	**3.91E-08**	**MOGAT2**	
PDFF	**12**	**3685100**	**rs1985912**	**intron_variant**	**G**	**A**	**0.326**	**−0.043**	**0.008**	**2.67E-08**	**PRMT8**	
PDFF (WFM)	**12**	**124476873**	**rs12833624**	**intron_variant**	**T**	**C**	**0.337**	**−0.039**	**0.006**	**3.37E-10**	**FAM101A**	**1.01E-06**
PDFF (VAT)	**17**	**7116853**	**rs446994**	**intron_variant**	**C**	**A**	**0.422**	**0.033**	**0.006**	**3.43E-09**	**DLG4**	**1.04E-08**
PDFF	17	17974014	rs11439486	downstream_gene_variant	T	TA	0.330	0.046	0.008	7.63E-09	GID4	2.19E-07
PDFF (VAT)	**17**	**64210580**	**rs1801689**	**missense_variant**	**C**	**A**	**0.029**	**0.099**	**0.016**	**1.63E-09**	**APOH**	
PDFF (NA)	19	18229208	rs56252442	intron_variant	T	G	0.253	0.048	0.008	5.11E-09	MAST3	5.13E-07
PDFF (VAT)	19	19379549	rs58542926	missense_variant	T	C	0.075	0.292	0.011	4.7E-168	TM6SF2	5.00E-10
PDFF (VAT)	**19**	**33834096**	**rs73026242**	**downstream_gene_variant**	**G**	**A**	**0.072**	**0.060**	**0.011**	**3.59E-08**	**CEBPG**	
PDFF (BMI)	19	45411941	rs429358	missense_variant	C	T	0.151	−0.102	0.008	2.92E-35	APOE	1.67E-15
PDFF	**19**	**45830763**	**rs344790**	**upstream_gene_variant**	**A**	**C**	**0.421**	**0.043**	**0.007**	**2.68E-09**	**CKM**	
PDFF (VAT)	19	54671421	rs60204587	intron_variant	A	G	0.426	0.045	0.006	1.42E-15	MBOAT7	5.27E-15
PDFF (WFM)	**20**	**39142516**	**rs2207132**	**intergenic_variant**	**A**	**G**	**0.034**	**0.092**	**0.016**	**1.73E-08**	**MAFB**	
PDFF (VAT)	22	44324730	rs738408	synonymous_variant	T	C	0.214	0.237	0.007	1.7E-269	PNPLA3	1.38E-14
cT1 (BMI)	**1**	**15810346**	**rs12130283**	**intron_variant**	**C**	**T**	**0.484**	**0.045**	**0.007**	**5.33E-10**	**CELA2B**	**6.91E-08**
cT1 (BMI)	1	220100497	rs759359281	splice_polypyrimidine_tract_variant	C	CA	0.055	0.129	0.016	4.74E-15	SLC30A10	
cT1 (VAT)	4	103188709	rs13107325	missense_variant	T	C	0.070	0.556	0.015	< 4.94E-324	SLC39A8	2.01E-18
cT1 (VAT)	6	25878848	rs55925606	intron_variant	G	A	0.073	−0.149	0.015	1.07E-24	SLC17A3	3.32E-09
cT1 (VAT)	**8**	**9173209**	**rs7012637**	**intron_variant**	**A**	**G**	**0.472**	**0.043**	**0.007**	**9.39E-09**	**RP11-10A14.4**	
cT1 (BMI)	**8**	**18272881**	**rs1495741**	**regulatory_region_variant**	**G**	**A**	**0.219**	**−0.064**	**0.009**	**4.5E-13**	**NAT2**	
cT1 (NA)	**9**	**136149830**	**rs532436**	**intron_variant**	**A**	**G**	**0.183**	**0.079**	**0.010**	**7.47E-15**	**ABO**	
cT1 (WFM)	10	113921825	rs2792735	intron_variant	G	A	0.277	0.046	0.008	4.05E-08	GPAM	4.81E-07
cT1 VAT)	**11**	**61549025**	**rs174533**	**intron_variant**	**A**	**G**	**0.351**	**0.052**	**0.008**	**2.49E-11**	**MYRF**	**3.51E-07**
cT1 (NA)	**12**	**51511269**	**rs59372312**	**intron_variant**	**G**	**A**	**0.067**	**−0.088**	**0.016**	**2.33E-08**	**TFCP2**	
cT1 (BMI)	14	24572932	rs111723834	missense_variant	A	G	0.016	0.383	0.029	1.7E-39	PCK2	9.99E-08
cT1 (VAT)	**16**	**77423427**	**rs2454933**	**intron_variant**	**T**	**C**	**0.082**	**0.075**	**0.014**	**3.69E-08**	**ADAMTS18**	
cT1 (NA)	**18**	**59323966**	**18:59323966_CTT_C**	**intron_variant**	**C**	**CTT**	**0.287**	**−0.050**	**0.009**	**1.28E-08**	**CDH20**	
cT1 (WFM)	19	19379549	rs58542926	missense_variant	T	C	0.074	0.141	0.014	4.22E-23	TM6SF2	4.46E-07
cT1 (VAT)	**19**	**33890838**	**rs10406327**	**intron_variant**	**G**	**C**	**0.477**	**−0.042**	**0.007**	**1.24E-08**	**PEPD**	**1.84E-06**
cT1 (BMI)	19	49206172	rs516246	intron_variant	C	T	0.489	−0.053	0.007	3.67E-13	FUT2	1.00E-13
cT1 (VAT)	22	37469192	rs6000553	intron_variant	A	G	0.466	0.070	0.007	7.53E-21	TMPRSS6	4.81E-12
cT1 (BMI)	22	44324727	rs738409	missense_variant	G	C	0.214	0.120	0.009	3.2E-41	PNPLA3	2.85E-13

The association between common genetic variants and PDFF under different adiposity adjustments was performed using REGENIE adjusting for adiposity index, age, sex, age×sex, age^2^ and age^2^×sex, first 10 genomic principal components and array batch. Each adiposity adjustment has been shown in parentheses. Genomic *loci* in bold represent the novel *loci* identified in the present work. Locus column shows the nearest gene to the lead variant (from COJO analysis) at each locus. MAGMA column shows significant gene-based associations at each locus exceeding Bonferroni threshold (P < 2.65E-6). P-values were not corrected for multiple testing among 4 different models (unadjusted, adjusted for BMI, WFM and VAT). PDFF: proton density fat fraction, cT1: liver iron corrected T1, WFM: whole-body fat mass (kg/m2), VAT: visceral adipose tissue.

## Data Availability

All data associated with this study are present in the paper or the Supplementary Information. For UK Biobank, all individual-level phenotype/genotype data are accessible via a formal application to the UK Biobank http://www.ukbiobank.ac.uk. Owing to study participants’ privacy data protection, the RNA-seq data of the liver visceral adipose biopsies can only be made available on request to the corresponding authors for collaborative projects. The following online databases have been used: GWAS catalog, https://www.ebi.ac.uk/gwas/; baseline LD model: https://data.broadinstitute.org/alkesgroup/LDSCORE/. All codes and scripts used for analyses are available on request.

## References

[1] Sanyal A.J., Prospective Study of Outcomes in Adults with Nonalcoholic Fatty Liver Disease. NEngl JMed 385, 1559–1569 (2021). 10.1056/NEJMoa2029349 PMC8881985 34670043

[2] Pais R., Redheuil A., Cluzel P., Ratziu V. & Giral P Relationship Among Fatty Liver, Specific and Multiple-Site Atherosclerosis, and 10-Year Framingham Score. Hepatology 69, 1453–1463 (2019). 30125370 10.1002/hep.30223

[3] Anstee Q.M., Targher G. & Day C. P Progression of NAFLD to diabetes mellitus, cardiovascular disease or cirrhosis. Nat Rev Gastroenterol Hepatol 10, 330–344 (2013). 23507799 10.1038/nrgastro.2013.41

[4] Toh J.Z.K., A Meta-Analysis on the Global Prevalence, Risk factors and Screening of Coronary Heart Disease in Nonalcoholic Fatty Liver Disease. Clin Gastroenterol Hepatol 20, 2462–2473.e2410 (2022). 34560278 10.1016/j.cgh.2021.09.021

[5] Romeo S., Sanyal A. & Valenti L. Leveraging Human Genetics to Identify Potential New Treatments for Fatty Liver Disease. Cell Metab 31, 35–45 (2020). 31914377 10.1016/j.cmet.2019.12.002

[6] Mantovani A., Adverse effect of PNPLA3 p.I148M genetic variant on kidney function in middle-aged individuals with metabolic dysfunction. Aliment Pharmacol Ther 57, 1093–1102 (2023). 36947711 10.1111/apt.17477

[7] Sun D.Q., An international Delphi consensus statement on metabolic dysfunction-associated fatty liver disease and risk of chronic kidney disease. Hepatobiliary Surg Nutr 12, 386–403 (2023). 37351121 10.21037/hbsn-22-421 PMC10282675

[8] Chen Y, Genome-wide association meta-analysis identifies 17 loci associated with nonalcoholic fatty liver disease. Nat Genet(2023). 10.1038/s41588-023-01497-6 PMC10918428 37709864

[9] Vujkovic M., A multiancestry genome-wide association study of unexplained chronic ALT elevation as a proxy for nonalcoholic fatty liver disease with histological and radiological validation. Nat Genet 54, 761–771 (2022). 35654975 10.1038/s41588-022-01078-z PMC10024253

[10] Emdin C.A., A missense variant in Mitochondrial Amidoxime Reducing Component 1 gene and protection against liver disease. PLoS Genet 16, e1008629 (2020). 32282858 10.1371/journal.pgen.1008629 PMC7200007

[11] Miao Z., Identification of 90 NAFLD GWAS loci and establishment of NAFLD PRS and causal role of NAFLD in coronary artery disease. HGG Adv 3, 100056 (2022). 35047847 10.1016/j.xhgg.2021.100056 PMC8756520

[12] Jamialahmadi O., Exome-Wide Association Study on Alanine Aminotransferase Identifies Sequence Variants in the GPAM and APOE Associated With Fatty Liver Disease. Gastroenterology 160, 1634–1646.e1637 (2021). 33347879 10.1053/j.gastro.2020.12.023

[13] Romeo S., Genetic variation in PNPLA3 confers susceptibility to nonalcoholic fatty liver disease. Nat Genet 40, 1461–1465 (2008). 18820647 10.1038/ng.257 PMC2597056

[14] Romeo S., Morbid obesity exposes the association between PNPLA3 I148M (rs738409) and indices of hepatic injury in individuals of European descent. Int J Obes (Lond) 34, 190–194 (2010). 19844213 10.1038/ijo.2009.216

[15] Agrawal S., Inherited basis of visceral, abdominal subcutaneous and gluteofemoral fat depots. Nat Commun 13, 3771 (2022). 35773277 10.1038/s41467-022-30931-2 PMC9247093

[16] Mbatchou J., Computationally efficient whole-genome regression for quantitative and binary traits. Nat Genet 53, 1097–1103 (2021). 34017140 10.1038/s41588-021-00870-7

[17] Bulik-Sullivan B.K., LD Score regression distinguishes confounding from polygenicity in genome-wide association studies. Nat Genet 47, 291–295 (2015). 25642630 10.1038/ng.3211 PMC4495769

[18] Finucane H.K., Partitioning heritability by functional annotation using genome-wide association summary statistics. Nat Genet 47, 1228–1235 (2015). 26414678 10.1038/ng.3404 PMC4626285

[19] Gazal S., Linkage disequilibrium-dependent architecture of human complex traits shows action of negative selection. Nat Genet 49, 1421–1427 (2017) . 28892061 10.1038/ng.3954 PMC6133304

[20] Chang C.C., Second-generation PLINK: rising to the challenge of larger and richer datasets. GigaScience 4(2015). 10.1186/s13742-015-0047-8 PMC4342193 25722852

[21] Yang J., Conditional and joint multiple-SNP analysis of GWAS summary statistics identifies additional variants influencing complex traits. Nat Genet 44, 369–375, S361–363 (2012). 22426310 10.1038/ng.2213 PMC3593158

[22] Kanai M., Genetic analysis of quantitative traits in the Japanese population links cell types to complex human diseases. Nat Genet 50, 390–400 (2018) . 29403010 10.1038/s41588-018-0047-6

[23] Buniello A., The NHGRI-EBI GWAS Catalog of published genome-wide association studies, targeted arrays and summary statistics 2019. Nucleic Acids Res 47, D1005–D1012 (2019). 30445434 10.1093/nar/gky1120 PMC6323933

[24] Weissbrod O., Functionally informed fine-mapping and polygenic localization of complex trait heritability. Nat Genet 52, 1355–1363 (2020). 33199916 10.1038/s41588-020-00735-5 PMC7710571

[25] Wang G., Sarkar A., Carbonetto P & Stephens M. A simple new approach to variable selection in regression, with application to genetic fine mapping. Journal of the Royal Statistical Society: Series B (Statistical Methodology) 82, 1273–1300 (2020). 37220626 10.1111/rssb.12388 PMC10201948

[26] Machiela M.J. & Chanock S.J. LDlink: a web-based application for exploring population-specific haplotype structure and linking correlated alleles of possible functional variants. Bioinformatics 31, 3555–3557 (2015). 26139635 10.1093/bioinformatics/btv402 PMC4626747

[27] Kerimov N., A compendium of uniformly processed human gene expression and splicing quantitative trait loci. Nat Genet 53, 1290–1299 (2021). 34493866 10.1038/s41588-021-00924-w PMC8423625

[28] Consortium G. The GTEx Consortium atlas of genetic regulatory effects across human tissues. Science 369, 1318–1330 (2020). 32913098 10.1126/science.aaz1776 PMC7737656

[29] Giambartolomei C., Bayesian test for colocalisation between pairs of genetic association studies using summary statistics. PLoS Genet 10, e1004383 (2014). 24830394 10.1371/journal.pgen.1004383 PMC4022491

[30] Watanabe K., Taskesen E., van Bochoven A. & Posthuma D. Functional mapping and annotation of genetic associations with FUMA. Nat Commun 8, 1826 (2017). 29184056 10.1038/s41467-017-01261-5 PMC5705698

[31] de Leeuw C.A., Mooij J.M., Heskes T. & Posthuma D. MAGMA: generalized gene-set analysis of GWAS data. PLoS Comput Biol 11, e1004219 (2015). 25885710 10.1371/journal.pcbi.1004219 PMC4401657

[32] Ren L., Genetic ablation of diabetes-associated gene Ccdc92 reduces obesity and insulin resistance in mice. iScience 26, 105769 (2023). 36594018 10.1016/j.isci.2022.105769 PMC9804112

[33] Choi K.M., Defective brown adipose tissue thermogenesis and impaired glucose metabolism in mice lacking Letmd1. Cell Rep 37, 110104 (2021). 34910916 10.1016/j.celrep.2021.110104 PMC12003058

[34] Dongiovanni P, Causal relationship of hepatic fat with liver damage and insulin resistance in nonalcoholic fatty liver. J Intern Med 283, 356–370 (2018). 29280273 10.1111/joim.12719 PMC5900872

[35] Dongiovanni P, Transmembrane 6 superfamily member 2 gene variant disentangles nonalcoholic steatohepatitis from cardiovascular disease. Hepatology 61, 506–514 (2015). 25251399 10.1002/hep.27490

[36] Tada H., Usui S., Sakata K., Takamura M. & Kawashiri M.A. Low-Density Lipoprotein Cholesterol Level cannot be too Low: Considerations from Clinical Trials, Human Genetics, and Biology. JAtheroscler Thromb 27, 489–498 (2020). 32350167 10.5551/jat.RV17040 PMC7355098

[37] Lauridsen B.K., Liver fat content, non-alcoholic fatty liver disease, and ischaemic heart disease: Mendelian randomization and meta-analysis of 279 013 individuals. Eur Heart J 39, 385–393 (2018). 29228164 10.1093/eurheartj/ehx662

[38] Stender S., Adiposity amplifies the genetic risk of fatty liver disease conferred by multiple loci. Nat Genet 49, 842–847 (2017). 28436986 10.1038/ng.3855 PMC5562020

[39] Sattar N., Forrest E. & Preiss D. Non-alcoholic fatty liver disease. BMJ 349, g4596 (2014). 25239614 10.1136/bmj.g4596 PMC4168663

[40] Pelusi S., Rare Pathogenic Variants Predispose to Hepatocellular Carcinoma in Nonalcoholic Fatty Liver Disease. Sci Rep 9, 3682 (2019). 30842500 10.1038/s41598-019-39998-2 PMC6403344

[41] Prill S., The TM6SF2 E167K genetic variant induces lipid biosynthesis and reduces apolipoprotein B secretion in human hepatic 3D spheroids. Sci Rep 9, 11585 (2019). 31406127 10.1038/s41598-019-47737-w PMC6690969

[42] Pirazzi C., Patatin-like phospholipase domain-containing 3 (PNPLA3) I148M (rs738409) affects hepatic VLDL secretion in humans and in vitro. J Hepatol 57, 1276–1282 (2012). 22878467 10.1016/j.jhep.2012.07.030

[43] Holmen O.L., Systematic evaluation of coding variation identifies a candidate causal variant in TM6SF2 influencing total cholesterol and myocardial infarction risk. Nat Genet 46, 345–351 (2014). 24633158 10.1038/ng.2926 PMC4169222

[44] Udler M.S., McCarthy M.I., Florez J.C. & Mahajan A. Genetic Risk Scores for Diabetes Diagnosis and Precision Medicine. Endocr Rev 40, 1500–1520 (2019) . 31322649 10.1210/er.2019-00088 PMC6760294

[45] Sudlow C., UK Biobank: An Open Access Resource for Identifying the Causes of a Wide Range of Complex Diseases of Middle and Old Age. PLOS Medicine 12, e1001779 (2015). 25826379 10.1371/journal.pmed.1001779 PMC4380465

[46] Bycroft C., The UK Biobank resource with deep phenotyping and genomic data. Nature 562, 203–209 (2018). 30305743 10.1038/s41586-018-0579-z PMC6786975

[47] Parisinos C.A., Genome-wide and Mendelian randomisation studies of liver MRI yield insights into the pathogenesis of steatohepatitis. J HepatoH 3, 241–251 (2020). 10.1016/j.jhep.2020.03.032 PMC7372222 32247823

[48] Jamialahmadi O., Tavaglione F., Rawshani A., Ljungman C. & Romeo S. Fatty liver disease, heart rate and cardiac remodelling: Evidence from the UK Biobank. Liver Int 43, 1247–1255 (2023). 36883784 10.1111/liv.15556

[49] Bulik-Sullivan B., An atlas of genetic correlations across human diseases and traits. Nat Genet 47, 1236–1241 (2015). 26414676 10.1038/ng.3406 PMC4797329

[50] Krzywinski M., Circos: an information aesthetic for comparative genomics. Genome Res 19, 1639–1645 (2009). 19541911 10.1101/gr.092759.109 PMC2752132

[51] Gazal S., Functional architecture of low-frequency variants highlights strength of negative selection across coding and non-coding annotations. Nat Genet 50, 1600–1607 (2018). 30297966 10.1038/s41588-018-0231-8 PMC6236676

[52] Lawrence M., Gentleman R. & Carey V rtracklayer: an R package for interfacing with genome browsers. Bioinformatics 25, 1841–1842 (2009). 19468054 10.1093/bioinformatics/btp328 PMC2705236

[53] Wallace C. A more accurate method for colocalisation analysis allowing for multiple causal variants. PLoS Genet 17, e1009440 (2021). 34587156 10.1371/journal.pgen.1009440 PMC8504726

[54] Cunningham F., Ensembl 2022. Nucleic Acids Res 50, D988–D995 (2022). 34791404 10.1093/nar/gkab1049 PMC8728283

[55] Ghoussaini M., Open Targets Genetics: systematic identification of trait-associated genes using large-scale genetics and functional genomics. Nucleic Acids Res 49, D1311–D1320 (2021). 33045747 10.1093/nar/gkaa840 PMC7778936

[56] Xie Z., Gene Set Knowledge Discovery with Enrichr. Curr Protoc 1, e90 (2021). 33780170 10.1002/cpz1.90 PMC8152575

[57] Lachmann A., Massive mining of publicly available RNA-seq data from human and mouse. Nat Commun 9, 1366 (2018). 29636450 10.1038/s41467-018-03751-6 PMC5893633

[58] Gillespie M., The reactome pathway knowledgebase 2022. Nucleic Acids Res 50, D687–D692 (2022). 34788843 10.1093/nar/gkab1028 PMC8689983

[59] de Mutsert R., The Netherlands Epidemiology of Obesity (NEO) study: study design and data collection. Eur J Epidemiol 28, 513–523 (2013). 23576214 10.1007/s10654-013-9801-3

[60] Valenti L., Definition of Healthy Ranges for Alanine Aminotransferase Levels: A 2021 Update. Hepatol Commun 5, 1824–1832 (2021). 34520121 10.1002/hep4.1794 PMC8557310

[61] Ellinghaus D., Genomewide Association Study of Severe Covid-19 with Respiratory Failure. N Engl J Med 383, 1522–1534 (2020). 32558485 10.1056/NEJMoa2020283 PMC7315890

[62] Tavaglione F., Accuracy of Controlled Attenuation Parameter for Assessing Liver Steatosis in Individuals With Morbid Obesity Before Bariatric Surgery. Liver Int (2021). 10.1111/liv.15127 34890093

[63] Dobin A., STAR: ultrafast universal RNA-seq aligner. Bioinformatics 29, 15–21 (2013). 23104886 10.1093/bioinformatics/bts635 PMC3530905

[64] Bolger A.M., Lohse M. & Usadel B. Trimmomatic: a flexible trimmer for Illumina sequence data. Bioinformatics 30, 2114–2120 (2014). 24695404 10.1093/bioinformatics/btu170 PMC4103590

[65] Li B. & Dewey C.N. RSEM: accurate transcript quantification from RNA-Seq data with or without a reference genome. BMC Bioinformatics 12, 323 (2011). 21816040 10.1186/1471-2105-12-323 PMC3163565

[66] Love M.I., Huber W. & Anders S. Moderated estimation of fold change and dispersion for RNA-seq data with DESeq2. Genome Biol 15, 550 (2014). 25516281 10.1186/s13059-014-0550-8 PMC4302049

[67] Leek J.T., Johnson W.E., Parker H.S., Jaffe A.E. & Storey J.D. The sva package for removing batch effects and other unwanted variation in high-throughput experiments. Bioinformatics 28, 882–883 (2012). 22257669 10.1093/bioinformatics/bts034 PMC3307112

